# A derivative of 3-(1,3-diarylallylidene)oxindoles inhibits dextran sulfate sodium-induced colitis in mice

**DOI:** 10.1007/s43440-024-00616-2

**Published:** 2024-06-25

**Authors:** Young-Jin Jeong, Hae-Ri Lee, Sun-Ae Park, Joong-Woon Lee, Lee Kyung Kim, Hee Jung Kim, Jae Hong Seo, Tae-Hwe Heo

**Affiliations:** 1 Laboratory of Pharmacoimmunology, Integrated Research Institute of Pharmaceutical Sciences, BK21 FOUR Team for Advanced Program for SmartPharma Leaders, College of Pharmacy, The Catholic University of Korea, NP512, Hall of Cardinal Jin-Suk Cheong, 43 Jibong-Ro, Bucheon-Si, Gyeonggi‑do 14662 Republic of Korea; 2 Laboratory of Pharmaceutical Manufacturing Chemistry, Integrated Research Institute of Pharmaceutical Sciences, College of Pharmacy, The Catholic University of Korea, 43 Jibong-Ro, Bucheon‑si, Gyeonggi‑do 14662 Republic of Korea

**Keywords:** Colitis, Intestinal barrier, Tight junction, gp130, STAT, Oxindole

## Abstract

**Background:**

IA-0130 is a derivative of 3-(1,3-diarylallylidene)oxindoles, which is a selective estrogen receptor modulator (SERM). A previous study demonstrated that SERM exhibits anti-inflammatory effects on colitis by promoting the anti-inflammatory phenotype of monocytes in murine colitis. However, the therapeutic effects of oxindole on colitis remain unknown. Therefore, we evaluated the efficacy of IA-0130 on dextran sulfate sodium (DSS)-induced mouse colitis.

**Methods:**

The DSS-induced colitis mouse model was established by administration of 2.5% DSS for 5 days. Mice were orally administered with IA-0130 (0.01 mg/kg or 0.1 mg/kg) or cyclosporin A (CsA; 30 mg/kg). Body weight, disease activity index score and colon length of mice were calculated and histological features of mouse colonic tissues were analyzed using hematoxylin and eosin staining. The expression of inflammatory cytokines and tight junction (TJ) proteins were analyzed using quantitative real-time PCR and enzyme-linked immunosorbent assay. The expression of interleukin-6 (IL-6) signaling molecules in colonic tissues were investigated using Western blotting and immunohistochemistry (IHC).

**Results:**

IA-0130 (0.1 mg/kg) and CsA (30 mg/kg) prevented colitis symptom, including weight loss, bleeding, colon shortening, and expression of pro-inflammatory cytokines in colon tissues. IA-0130 treatment regulated the mouse intestinal barrier permeability and inhibited abnormal TJ protein expression. IA-0130 down-regulated IL-6 expression and prevented the phosphorylation of signaling molecules in colonic tissues.

**Conclusions:**

This study demonstrated that IA-0130 suppressed colitis progression by inhibiting the gp130 signaling pathway and expression of pro-inflammatory cytokines, and maintaining TJ integrity.

**Supplementary Information:**

The online version contains supplementary material available at 10.1007/s43440-024-00616-2.

## Introduction

Inflammatory bowel disease (IBD) is an autoimmune disease characterized by chronic inflammation of the gastrointestinal tract and can be classified into Crohn’s disease (CD) and ulcerative colitis (UC) [1]. Patients with IBD exhibit weight loss, fatigue, and a rapid loss of their quality of life [2]. Its causes are complex and include abnormal immune responses, epithelial barrier defects, and environmental and genetic factors [3].

The intestinal epithelial barrier regulates the exchange of nutrients and ions and maintains immune tolerance by preventing the invasion of pathogenic antigens [4]. These functions are performed by tight junction (TJ) proteins, which regulate the permeability of the intestinal epithelial barrier [5]. TJ proteins include occludin, junctional adhesion molecule A (JAM-A), zonular occludens-1 (ZO-1), and claudin-2 [6]. Occludin, JAM-A, and ZO-1 increase junction tightness, whereas claudin-2 regulates ion exchange via the pore pathway and increases barrier permeability [7]. Abnormal TJ protein expression induces dysregulation of intestinal barrier permeability, leading to antigen infiltration [8]. Immune cells recognize antigens and secrete various inflammatory cytokines [9].

Pro-inflammatory cytokines such as tumor necrosis factor-α (TNF-α), interleukin 1-β (IL-1β), IL-6, and IL-17A secreted from immune cells enhance intestinal inflammation development by activating antigen-presenting cells and T cells [10]. Inflammatory cytokine secretion by lamina propria dendritic cells and macrophage is elevated in the mucosa of patients with UC [11]. IL-6 binds to glycoprotein 130 (gp130), and activates Janus kinase (JAK) and signal transducer and activator of transcription (STAT) signaling in macrophages and T lymphocytes. STAT3 maintains the survival and proliferation of pathogenic T cells [12]. The gp130 signaling induces the recruitment and activation of neutrophils and macrophages, leading to acute inflammation in colitis [13]. Therefore, regulation of the excessive IL-6 signaling cascade is important for treating colitis.

The targeting of inflammatory cytokines is considered an effective strategy in IBD treatment [14, 15], which commonly uses TNF inhibitors [16]. Since a few patients do not respond to anti-TNF therapies [17], IL-6 signaling inhibitors are used in alternative therapies [18, 19]. JAK inhibitor tofacitinib has therapeutic efficacy in patients with IBD and UC-like mouse models [20, 21]. In a previous study, a selective estrogen receptor modulator (SERM) named bazedoxifene was shown to alleviate colitis via nucleotide-binding oligomerization domain 2 mutations in a dextran sulfate sodium (DSS)-induced zebrafish CD model [22]. IA-0130 is a small-molecule derivative of 3-(1,3-diarylallylidene)oxindoles [23]. Oxindoles are heterogeneous aromatic natural compounds found in a variety of plants and mammalian animals [24]. Oxindole derivatives have shown anticancer, antibacterial, antioxidant, and antiviral activities, and have proven therapeutic effects on rheumatoid arthritis and tuberculosis diseases [25]. We previously screened a drug library to find hit compounds that inhibit IL-6 activity using HEK-Blue IL-6 cells expressing genes encoding human IL-6 and STAT. As a result, IA-0130 most effectively inhibited the proliferation of HEK-Blue IL-6 cells, and surface plasmon resonance analysis confirmed that IA-0130 specifically binds to gp130, not IL-6 or IL-6Rα, and IA-0130 inhibits ovarian cancer cell proliferation, migration, invasion, and tumor growth [26]. However, the anti-inflammatory effects of IA-0130 remain unknown. In this study, we aimed to investigate the efficacy of IA-0130 in suppressing DSS-induced murine colitis development.

## Materials and methods

### Chemicals and reagents

DSS (molecular weight = 36,000–50,000 Da, #1,60,110) was purchased from MP Biomedicals (Santa Ana, CA, USA). IA-0130 was provided by Prof. Jae Hong Seo of the Pharmaceutical Manufacturing Chemistry Laboratory at the Catholic University of Korea. Cyclosporin A (CsA, #30,024), dimethyl sulfoxide, methylcellulose, paraformaldehyde, fluorescein isothiocyanate (FITC)-conjugated dextran (4 kDa), chloroform, 3,3ʹ-diaminobenzidine (DAB) solution, and Tween-20 were purchased from Sigma-Aldrich (St. Louis, MO, USA). Phosphate-buffered saline (PBS) was purchased from Welgene (Gyeongsangbuk-do, Republic of Korea). Ethanol and isopropanol were purchased from DAEJUNG (Gyeonggi-do, Republic of Korea). Xylene was purchased from DUKSAN (Gyeonggi-do, Republic of Korea). Paraffin pastilles and Immobilon®-P transfer membrane were purchased from Merck Millipore (Burlington, MA, USA). EASAYSTAIN Harris hematoxylin was purchased from YD Diagnostics (Gyeonggi-do, Republic of Korea). Eosin Y solution was purchased from MUTO PURE Chemicals (Tokyo, Japan). The mounting solution and SuperSignal West Femto Maximum Sensitivity Substrate were purchased from Thermo Fisher Scientific (Waltham, MA, USA). IL-1β, IL-6, IL-17A, and TNF-α cytokine enzyme-linked immunosorbent assay (ELISA) kits were purchased from BioLegend (San Diego, CA, USA). Fetal bovine serum (FBS) was purchased from Corning (Corning, NY, USA). Bovine serum albumin (BSA) was purchased from BOVOGEN (Melbourne, Australia). The 3,3′,5,5′-tetramethylbenzidine (TMB) substrate solution was purchased from Surmodics (Eden Prairie, MN, USA). TRIzol reagent, NuPAGE sodium dodecyl sulfate–polyacrylamide gel electrophoresis (SDS-PAGE) gradient loading gel (4–12%), and (3-(N-morpholino)propanesulfonic acid) were obtained from Invitrogen (Waltham, MA, USA). Diethyl pyrocarbonate-treated water was purchased from Biosesang (Gyeonggi-do, Republic of Korea). A cDNA synthesis kit and TB Green Premix Ex Taq II were obtained from TAKARA Bio (Shiga, Japan). Halt™ protease and phosphatase inhibitor cocktail (Thermo Fisher Scientific) was mixed at a 1/100 ratio with radioimmunoprecipitation assay (RIPA) buffer (Biosesang). Tris-buffered saline (TBS) (pH 7.5) was prepared by mixing 200 mM Tris (Duchefa Biochemie, Haarlem, Netherlands) and 1.5 M sodium chloride (Biosesang). Skim milk was purchased from BD Biosciences (Franklin Lakes, NJ, USA). Primary antibodies anti-p-gp130 (Biorbyt, Cambridge, UK, #orb449135) and anti-p-STAT3 (Abcam, #ab76315) were used in immunohistochemistry (IHC) analysis. Western blotting primary antibodies against IL-6 (#ab6672) was obtained from Abcam (Cambridge, United Kingdom). Primary antibodies against JAM-1 (#36-1700), ZO-1 (#ZO1-1A12), claudin-2 (#32-5600), phosphorylated (p)-gp130 (#PA5-105,780), and p-JAK2 (#44-426G) were purchased from Invitrogen. Primary antibodies against Occludin (#91,131), gp130 (#3732), JAK2 (#3230), p-STAT3 (#9145), STAT3 (#9132), β-actin (#4967), and HRP-conjugated anti-rabbit IgG (#7074) were obtained from Cell Signaling Technology (Danvers, MA, USA).

### Animals

Male C57BL/6 J mice (weight, 19–22 g; age, 6 weeks) were purchased from Orient Bio (Gyeonggi-do, Republic of Korea). Mice were monitored daily during the one-week adaptation period and housed under 12-h light and 12-h dark cycle (lights on from 07:00 to 19:00) with a constant temperature (22–24 ºC) and humidity (50–60%). Animal experiments were conducted according to internationally accepted standards and approved protocols (approval number: CUK-IACUC-2022-015-01, permission code, March 2022) by the Institutional Animal Care and Use Committee at the Songsim campus of The Catholic University of Korea (Bucheon-si, Gyeonggi-do, Republic of Korea).

### DSS-induced colitis mouse model

Male C57BL/6 J mice (21–24 g; age, 6 weeks) were randomly divided into five groups: Normal, DSS control, IA-0130 0.01 mg/kg treated group, IA-0130 0.1 mg/kg treated group, and CsA treated group (n = 6/group). Mice were administered 2.5% DSS (w/v) dissolved in drinking water for a total of 5 days. After 5 days, drinking water was replaced with regular tap water and supplied until the endpoint. Drinking water without DSS was provided to the control group. Oral administration of IA-0130 (0.01 mg/kg or 0.1 mg/kg) or CsA (30 mg/kg) was conducted simultaneously with DSS treatment and continued daily until the end of the experiment. Body weight and colitis symptoms such as diarrhea and bleeding were measured daily. The disease activity index (DAI) was determined based on body weight loss, stool consistency, and bleeding. The DAI scores were calculated using previous guidelines [27] described in Table S1.

DAI score = (Weight loss rate score + Stool consistency score + Occult blood degree score)/3.

After 9 d, the mice were euthanized, and the colon length was measured. Colon tissue samples were collected for subsequent analysis.

### Histopathological analyses

Colon tissues were extracted from C57BL/6 mice (n = 6/group) and washed using ice-cold PBS. The tissues were cut into 1–1.5 cm pieces and fixed with 4% paraformaldehyde in PBS at 4 °C for 12 h. After washing using PBS (10 min, 2 times), the tissues were dehydrated by treatment with ethanol at 90%, 95%, and 100% concentrations for 1 h each and xylene for 1 h. Colonic tissues were embedded with paraffin at 65 °C overnight and transferred to fresh paraffin for 2 h. Paraffin-embedded blocks were sectioned (8 µm) using HistoCore AUTOCUT microtome (Leica) on a total of 3 slides by early, mid, and late stages according to the sectioning stage per mouse tissue and dried overnight in 37 °C incubator. Sections were rehydrated by treatment with ethanol at 100% and 95% concentrations for 2 min each and xylene. To evaluate histological features of the mucosa, sections were stained with hematoxylin for 3 min and washed with running water, and were treated with 95% ethanol for 1 min. And then, the sections were treated with eosin for 50 s and dehydrated by treatment with ethanol at 95%, 100%, and 100% concentrations for 2 min each and xylene. The tissues were covered with the mounting solution.

### Immunohistochemistry

The tissue sections were hydrated using xylene and ethanol for 2 min per step. After washing with distilled water, the sections were treated with blocking buffer (10% FBS in 1 × PBS) in a humidified chamber at room temperature for 30 min. To assess the protein expression of IL-6 signaling molecules in mouse colonic tissue, the sections were treated with primary antibodies (1:200 dilution), including anti-p-gp130 and anti-p-STAT3, which were diluted in PBS containing 1% BSA, and incubated in a humidified chamber at 4 °C overnight. After washing using PBS (5 min, 3 times), the slides were treated with HRP-conjugated secondary antibodies diluted in PBS containing 1% BSA at room temperature for 1 h. Subsequently, the slides were treated with DAB solution (1:500 dilutions) for 2–5 min to confirm proper color development. After washing with PBS, the sections were dehydrated by treatment with ethanol at 95%, 100%, and 100% concentrations for 2 min each and xylene. The mounted sections were mounted using a mounting solution and dried at room temperature for 24 h.

### Histological image analysis

Histopathological analysis of H&E staining and IHC was performed by Orient GENIA (Gyeonggi-do, Republic of Korea) based on the reference guidelines [28]. The distal colonic region of the mouse colon was observed using a microscope (Leica, DM300) and Microscope Camera (Nikon, Digital Sight 50 M) in three sections per mouse. Mounted slides were scanned using an APERIO CS2 slide scanner (Leica, Wetzlar, Germany) at 200X (H&E staining) and 50X (IHC) magnifications. In histological analysis of H&E staining, the cytoplasm was stained with eosin to measure loss of intestinal epithelial layer, destruction of crypt structures, and depletion of goblet cells. Additionally, the nuclei of cells in the mucosal layer and submucosa, such as neutrophils and macrophages, were stained with hematoxylin to measure the number of infiltrated immune cells. The level of loss of epithelium and crypt damage was calculated as the ratio of the area of ​​the inflamed site where pathological features were identified and the level of goblet cell depletion and infiltration of inflammatory cells was evaluated by measuring the number of cells per area. Histopathological evaluation criteria for H&E staining are described in Table S2. The IHC analysis results were calculated by measuring the area ratio of the positive area stained with IHC antibody to the total area, and the differences between groups were compared and presented in a graph. The scan files were analyzed using ScanScope Virtual Slide software (Leica).

### ELISA

Antibodies against mouse IL-1β, IL-6, IL-17A, and TNF-α were diluted in 1 × PBS and added to Immuno 96-well plates (Thermo Fisher Scientific). Coated plates were incubated at 4 °C overnight. The plates were treated with 200 µl/well blocking buffer (1% BSA dissolved in 1 × PBS) and incubated at room temperature for 1 h. Mouse IL-1β, IL-6, IL-17A, and TNF-α ELISA standard proteins (BioLegend) and mouse colonic tissue homogenates were added to the plates and incubated for 2 h at room temperature. After incubation for 2 h, the detection antibody diluted in blocking buffer was incubated at room temperature for 1 h, and the avidin–horseradish peroxidase (HRP)-conjugated secondary antibody (BioLegend) was added for 30 min. Sufficient color development was confirmed after treatment with TMB and incubation at room temperature. The reaction was stopped using 2N HCl. Optical density was measured at 450 nm using a microplate reader (BioTek, Winooski, VT, USA). Standard curves with known protein concentration were created using ELISA standard mouse proteins for quantitative analysis.

### Intestinal permeability assay

FITC-conjugated dextran was dissolved in sterilized PBS at a concentration of 80 mg/mL. Mice were fasted for 4 h before FITC-dextran injection. Mice were orally administered with 100 μL FITC-dextran. After 4 h, mouse blood was collected and centrifuged at 4000 rpm for 15 min. Plasma samples were collected into clear Eppendorf tubes (Eppendorf, Hamburg, Germany) and placed in the dark at 4 °C. Standard FITC-dextran samples were diluted with PBS at the range of 0 to 100 μg/mL and transferred to a black 96-well plate (Corning). Fluorescence was measured at an emission wavelength of 528 nm and an excitation wavelength of 485 nm using a BioTek Synergy Neo2 microplate reader (Agilent Technologies, Santa Clara, CA, USA).

### RNA extraction and quantitative real-time polymerase chain reaction

Total RNA was extracted from colonic tissues using TRIzol reagent, and 1 μg of RNA was used for cDNA synthesis using the PrimeScript™ RT Master Mix cDNA synthesis kit (TAKARA Bio). Quantitative real-time polymerase chain reaction (qRT-PCR) was performed using TB Green Premix Ex Taq II, according to the manufacturer’s protocol. Briefly, initial denaturation was performed at 95 °C for 30 s and thermal cycling was repeated in a total of 40 cycles at 95 °C for 5 s and 60 °C for 30 s per cycle. The relative mRNA expression levels were normalized to that of β-actin. The primers used for RT-PCR are summarized in Table 1.

**Table 1:** qRT-PCR primer sequences

Gene	Primer sequence (5'-3ʹ)	
	Forward	Reverse
*occludin*	TGAAAGTCCACCTCCTTACAGA	CCGGATAAAAAGAGTACGCTGG
*jam-a*	TTGACCTGCACCTACTCTGG	GTAGTTCTGGCCACCTTCCT
*zo-1*	GCTTTAGCGAACAGAAGGAGC	TTCATTTTTCCGAGACTTCACCA
*cldn2*	CCCCAACCTTGCATGGATCT	ACAATGCTGGCACCGACATA
*il-1β*	ATGGCAACTGTTCCTGAACTCAACT	CAGGACAGGTATAGATTCTTTCCTTT
*il-6*	AGGATACCACTCCCAACAGACCT	CAAGTGCATCATCGTTGTTCATAC
*il-17a*	CCGCAATGAAGACCCTGATA	CTCGACCCTGAAAGTGAAGG
*tnf-α*	CATCTTCTCAAAATTCGAGTGACAA	TGGGAGTAGACAAGGTACAACCC
*β-actin*	TAGGCGGACTGTTACTGAGC	GCCTTCACCGTTCCAGTTTT

### Protein extraction and Western blot analysis

Mouse colon tissues were lysed using RIPA lysis buffer and proteins in lysates were quantified using a Pierce bicinchoninic acid assay kit (Thermo Fisher Scientific). Protein extracts (100 µg) were separated via SDS-PAGE and transferred to membranes. Membranes were incubated in blocking buffer (3% BSA in 1 × TBS buffer and/or 5% skim milk in 1 × TBS buffer) for 1 h and treated overnight with primary antibodies against IL-6, JAM-1, ZO-1, occludin, claudin-2, phosphorylated (p)-gp130, p-JAK2, p-STAT3, gp130, JAK2, STAT3 (1:1,000 dilutions), and β-actin (1:4,000 dilutions). After incubation, the membranes were treated with HRP-conjugated anti-rabbit IgG (1:4,000 dilutions) for 1 h. In all steps, 3–5 washes of 5 min each were performed using wash buffer (0.5% Tween-20 in 1 × TBS buffer). SuperSignal West Femto maximum sensitivity substrate (Thermo Fisher Scientific) was used for detection, and signal bands were analyzed using the ChemiDoc XRS gel imaging system (BioRad Laboratories, Hercules, CA, USA). Band densitometry was performed using ImageJ software.

### Statistical analysis

Statistical analysis of the data was performed using GraphPad Prism 8 (GraphPad Software, San Diego, CA, USA). Normality of data was tested for normal distribution using D'Agostino-Pearson Test. Data showing a normal distribution were analyzed using two-way ANOVA, and data not following a normal distribution were analyzed using the Kruskal–Wallis test. Statistical analysis of body weight change and DAI score of mice data were performed using two-way ANOVA (independent variables: time and treatment) followed by the Dunnett’s multiple comparisons test to analyze the differences of individual values between the groups, and the data are presented as the mean and standard error of the mean. Non-parametric results of mouse colon length, histological score, RT-qPCR and ELISA, FITC-dextran assay, Western blotting, IHC data were analyzed by Kruskal–Wallis test followed by the Dunn’s multiple comparisons test and the data are presented as the median with interquartile range. *p—*value < 0.05 was considered statistically significant.

## Results

### *IA*-0130 ameliorated DSS-induced mouse colitis

**Fig. 1:**
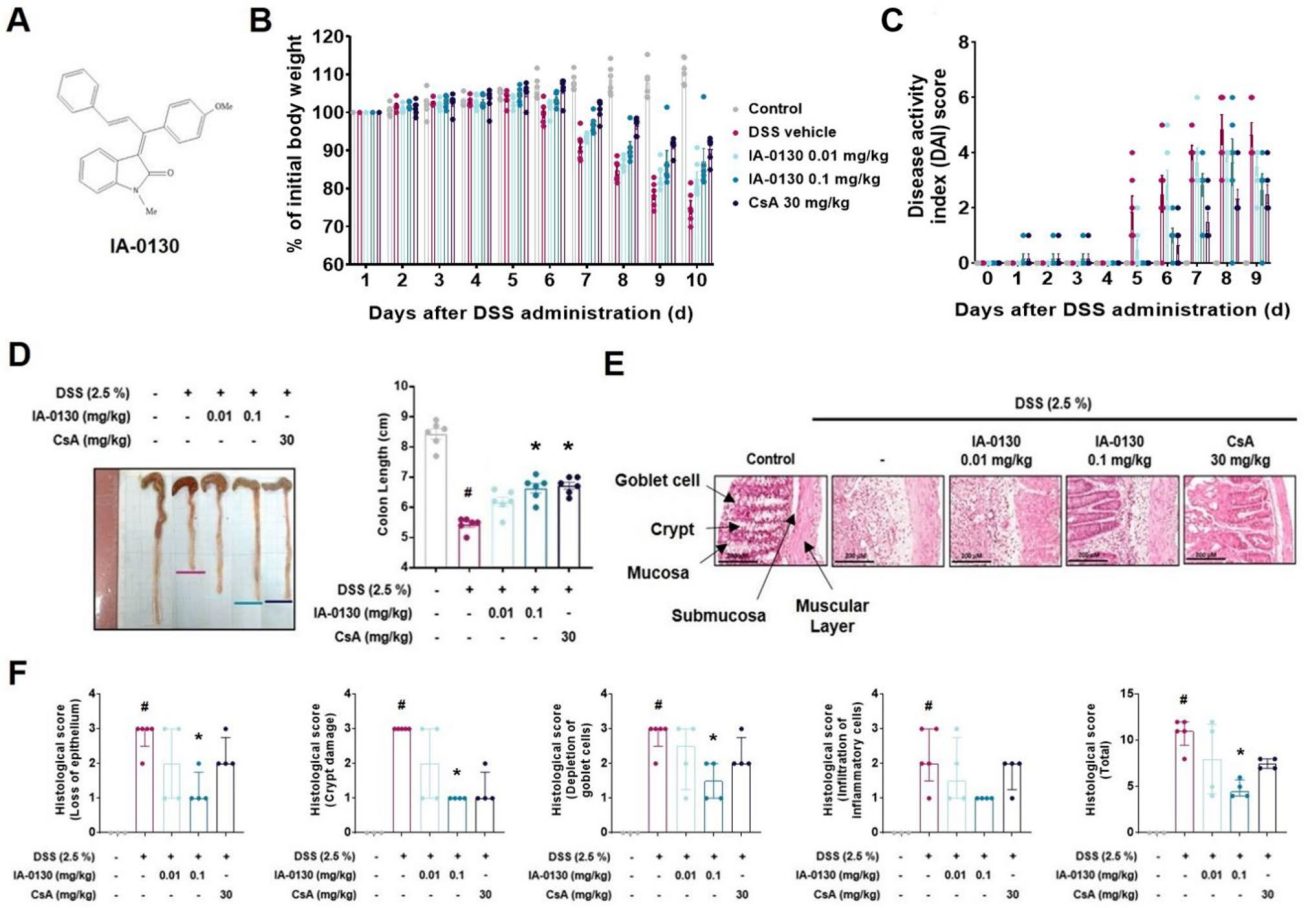
IA-0130 ameliorated DSS-induced colitis. C57BL/6 mice (n = 6/group) were treated with 2.5% DSS in their drinking water for 5 days and given normal drinking water for another 4 days. Chemical structure of IA-0130 (A). IA-0130 (0.01 or 0.1 mg/kg) or CsA (30 mg/kg) were orally administered everyday for 9 days. The percentage (%) of change of initial body weight (B), DAI score (C), and colon length (D) were measured. Colon sections of mice treated with DSS alone, DSS + IA-0130 or DSS + CsA were stained with hematoxylin and eosin (H&E) and (scale bar = 200 µm) scanned using APERIO CS2 slide scanner (Leica) (E). The histological scoring of H&E staining was measured based on the criteria: loss of epithelium, crypt damage, depletion of goblet cells, infiltration of inflammatory cells (F). The data of body weight change and DAI score of mice were analyzed by two-way ANOVA followed by the Dunnett’s multiple comparisons test and the data are presented as mean and standard error of the mean. The data of colon length and histological analysis were analyzed using Kruskal–Wallis test followed by the Dunn’s multiple comparisons test and the data are presented as the median with interquartile range. # *p* < 0.05 vs. control group, * p < 0.05, ** p < 0.01, *** *p* < 0.005 vs. DSS group. CsA, cyclosporin A; DAI, disease activity index; DSS, dextran sulfate sodium

To investigate whether IA-0130 had an inhibitory effect on colitis, we established a mouse model of DSS-induced colitis and observed colitis symptoms. Mice were orally administered with IA-0130, a 3-(1,3-diarylallylidene)oxindoles derivative (Fig. 1A), at a dose of 0.01 and 0.1 mg/kg and 30 mg/kg of CsA. As a result, the significant effect on time of analysis (F_2.032,40.64_ = 249.3, *p* < 0.001) and treatment (F_3,20_ = 12.10, *p* < 0.001) was confirmed. Additionally, the body weight loss was significantly inhibited by treatment of 0.1 mg/kg of IA-0130 (F_3,20_ = 12.10, *p* = 0.04) compared to that in the DSS vehicle group (Fig. 1B). The mice treated with 0.01 mg/kg IA-0130 exhibited a slower body weight loss than DSS vehicle group, but there was no statistical significance (F_3,20_ = 12.10 and *p* = 0.05). The severity of colitis symptoms, such as weight loss, consistency of stools, and bleeding, was evaluated using the DAI score. There was significant effect on the time of analysis (F_3.366,67.32_ = 109.2, *p* < 0.001) and treatment (F_3,20_ = 8.684, *p* < 0.001). DAI score was lower in the 0.1 mg/kg IA-0130 treated group (F_3,20_ = 8.684, *p* = 0.04) and CsA treated groups (F_3,20_ = 8.684, *p* = 0.007) than the DSS group (Fig. 1C). Furthermore, we found that IA-0130 (H_3_ = 23.95, N_1-5_ = 6, p = 0.04, Kruskal–Wallis test) and CsA (H_3_ = 23.95, N_1-5_ = 6, p = 0.02, Kruskal–Wallis test) significantly suppressed DSS-induced shortening of the mouse colon (Fig. 1D). The results of hematoxylin and eosin staining showed that the administration of 0.1 mg/kg of IA-0130 significantly inhibited the histological features of colitis (H_3_ = 23.53, N_1-5_ = 6, p = 0.04, Kruskal–Wallis test for the total score), such as loss of epithelium, infiltration of inflammatory cells, and preserved the crypt structures and goblet cells (Fig. 1E and 1F). The histological pathology inhibition effect was confirmed in the mouse group administered CsA at a concentration of 30 mg/kg (H_3_ = 23.53, N_1-5_ = 6, p = 0.54, Kruskal–Wallis test for the total score), but this was confirmed to be more effectively inhibited in the group administered IA-0130 at a concentration of 0.1 mg/kg (H_3_ = 23.53, N_1-5_ = 6, p = 0.04, Kruskal–Wallis test for the total score). Collectively, these results demonstrated that IA-0130 effectively prevented the development of DSS-induced mouse colitis.

### *IA*-0130 suppressed pro-inflammatory cytokines expression in mouse colonic tissues

**Fig. 2:**
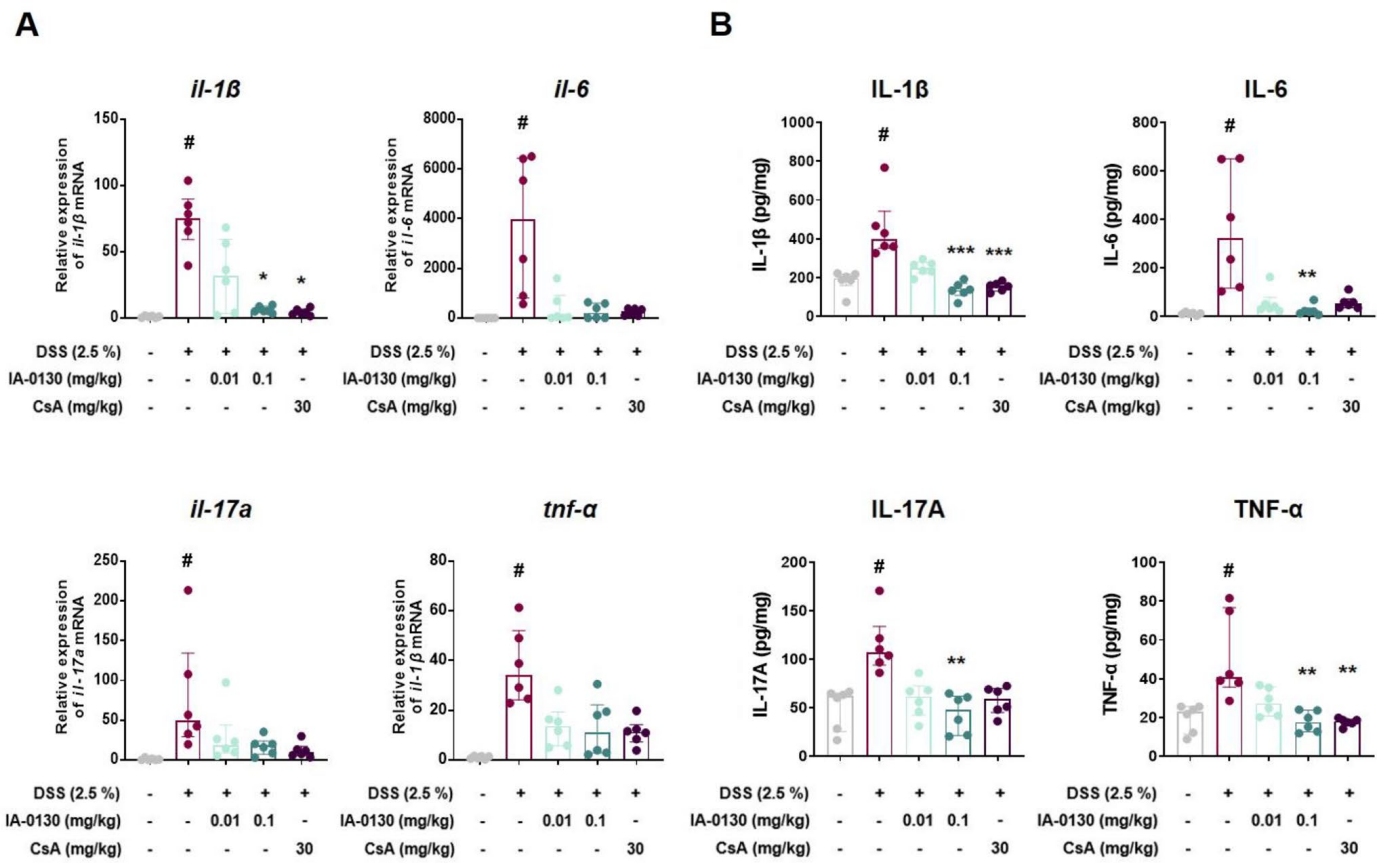
IA-0130 suppressed pro-inflammatory cytokine expression in mouse colon tissues. The mRNA levels (A) and the protein expression (B) of IL-1β, IL-6, IL-17A, and TNF-α in the colon tissues of mice, n = 6/group. The data of RT-qPCR and ELISA were analyzed using Kruskal–Wallis test followed by the Dunn’s multiple comparisons test and the data are presented as the median with interquartile range. # *p* < 0.05 vs. control group, * *p* < 0.05, ** *p* < 0.01, *** *p* < 0.005 vs. DSS group. IL, interleukin; TNF, tumor necrosis factor

Subsequently, we assessed the efficacy of IA-0130 in maintaining pro-inflammatory cytokine homeostasis. As a result, the mRNA levels of pro-inflammatory cytokines were upregulated in inflamed mouse colon tissues. Treatment with 0.1 mg/kg IA-0130 down-regulated *il-1β* (H_3_ = 22.59, N_1-5_ = 6, p = 0.11, Kruskal–Wallis test), *il-6* (H_3_ = 20.88, N_1-5_ = 6, p = 0.11, Kruskal–Wallis test), *il-17a* (H_3_ = 19.78, N_1-5_ = 6, p = 0.29, Kruskal–Wallis test), and *tnf-α* (H_3_ = 20.96, N_1-5_ = 6, p = 0.11, Kruskal–Wallis test) cytokine mRNA levels (Fig. 2A). Similarly, ELISA results showed that the colonic tissues from mice treated with IA-0130 exhibited significantly lower protein expression of the IL-1β (H_3_ = 23.29, N_1-5_ = 6, p < 0.001, Kruskal–Wallis test), IL-6 (H_3_ = 22.41, N_1-5_ = 6, p = 0.003, Kruskal–Wallis test), IL-17A (H_3_ = 15.81, N_1-5_ = 6, p = 0.001, Kruskal–Wallis test), and TNF-α (H_3_ = 18.79, N_1-5_ = 6, p = 0.002, Kruskal–Wallis test) cytokines than the DSS vehicle groups (Fig. 2B). In conclusion, IA-0130 effectively inhibited the excessive increase of pro-inflammatory cytokines expression in inflamed mouse colons.

### *IA*-0130 regulated the intestinal barrier function in mouse colonic tissues

**Fig. 3:**
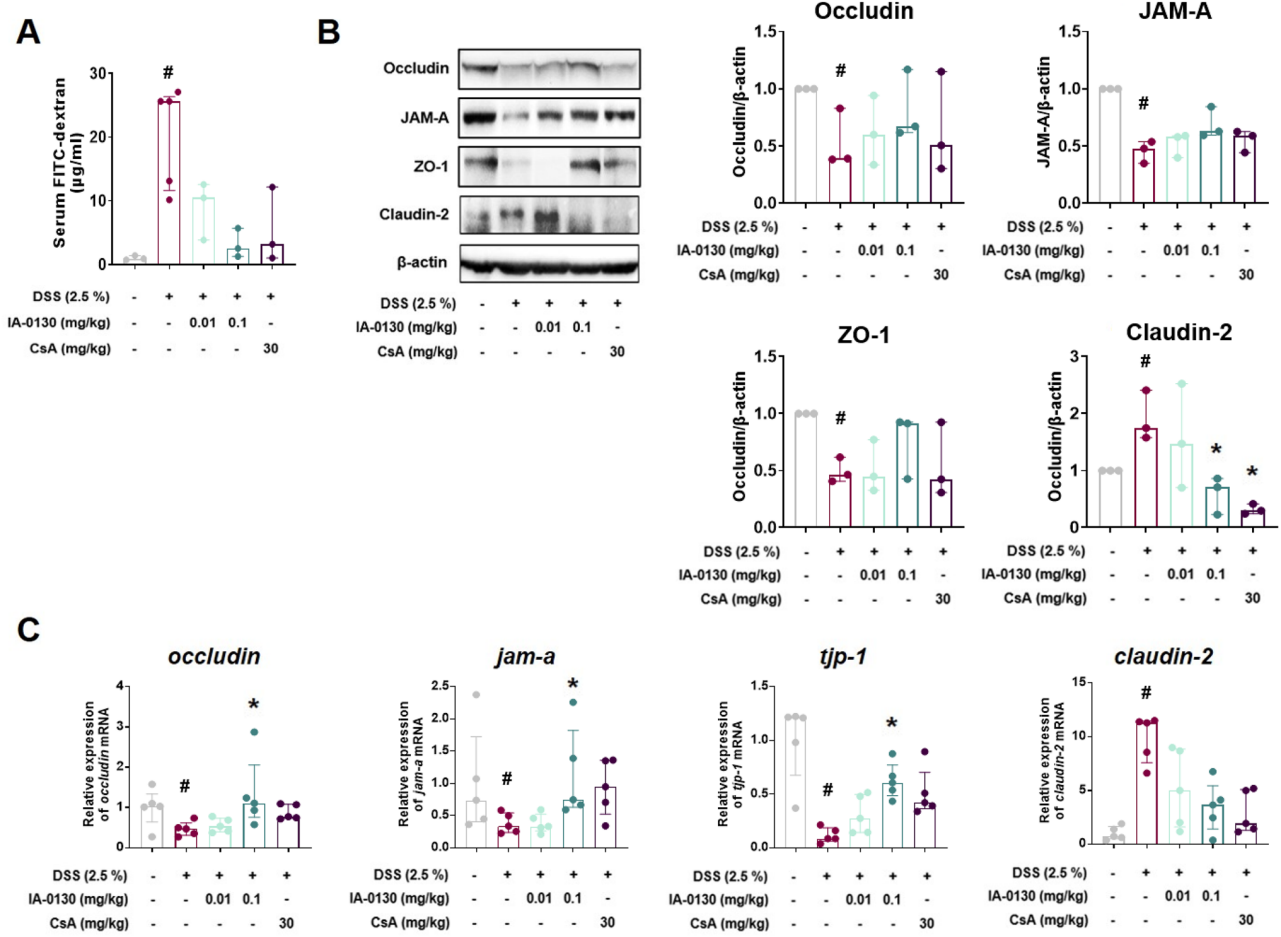
IA-0130 regulated intestinal barrier functions in mouse colon tissues. Levels of FITC-conjugated dextran in the serum of DSS-colitis mice, n = 3/group (A). Densitometric analysis of ZO-1, JAM-A, occludin, and claudin-2 expression in the colon tissues of mice (B) and the mRNA levels of *tjp-1*, *jam-a*, *occludin*, and *claudin-2* in the colon tissues of mice, n = 6/group (C). The data of FITC-dextran assay, Western blotting, and RT-qPCR were analyzed using Kruskal–Wallis test followed by the Dunn’s multiple comparisons test and the data are presented as the median with interquartile range. # *p* < 0.05 vs. control group, * *p* < 0.05, ** *p* < 0.01, *** *p* < 0.005 vs. DSS group. FITC, fluorescein isothiocyanate; JAM-A, junctional adhesion molecule-A; ZO-1, zonula occludens-1

The FITC-dextran 4 assay was performed to determine the intestinal barrier permeability by measuring the quantity of FITC-conjugated dextran leaking from the mouse colon into the blood. Dextran concentration in the serum of mice increased in the DSS vehicle group (Fig. 3A) but treatment with 0.1 mg/kg IA-0130 inhibited dextran permeation (H_3_ = 11.94, N_1-5_ = 3–5, p = 0.11, Kruskal–Wallis test). This result suggests that the administration of IA-0130 effectively down-regulated intestinal permeability in DSS-induced colitis. To determine how IA-0130 affects barrier function, we analyzed the expression of the TJ proteins, which are one of the critical factors determining intestinal barrier permeability. The expression of TJ proteins in mouse colon tissues was analyzed by Western blotting (Fig. 3B). The results showed that IA-0130 treatment prevented the reduction of occludin (H_3_ = 4.969, N_1-5_ = 3, p = 0.80, Kruskal–Wallis test), JAM-A (H_3_ = 11.15, N_1-5_ = 3, p = 0.18, Kruskal–Wallis test), and ZO-1 (H_3_ = 8.091, N_1-5_ = 3, p = 0.80, Kruskal–Wallis test) expression and inhibited the upregulation of claudin-2 expression (H_3_ = 10.21, N_1-5_ = 3, p = 0.04, Kruskal–Wallis test) by DSS-treatment. Furthermore, administration with 0.1 mg/kg IA-0130 improved the abnormal changes in *occludin* (H_3_ = 11.66, N_1-5_ = 6, p = 0.02, Kruskal–Wallis test), *jam-a* (H_3_ = 12.64, N_1-5_ = 6, p = 0.04, Kruskal–Wallis test), *tjp-1* (H_3_ = 16.45, N_1-5_ = 6, p = 0.01, Kruskal–Wallis test), and *claudin-2* (H_3_ = 14.59, N_1-5_ = 6, p = 0.13, Kruskal–Wallis test) mRNA levels (Fig. 3C). These results demonstrated that IA-0130 maintained intestinal barrier permeability by regulating TJ protein expression.

### *IA*-0130 inhibited the hyperactivation of IL-6 signaling in the mouse *colon*

**Fig. 4:**
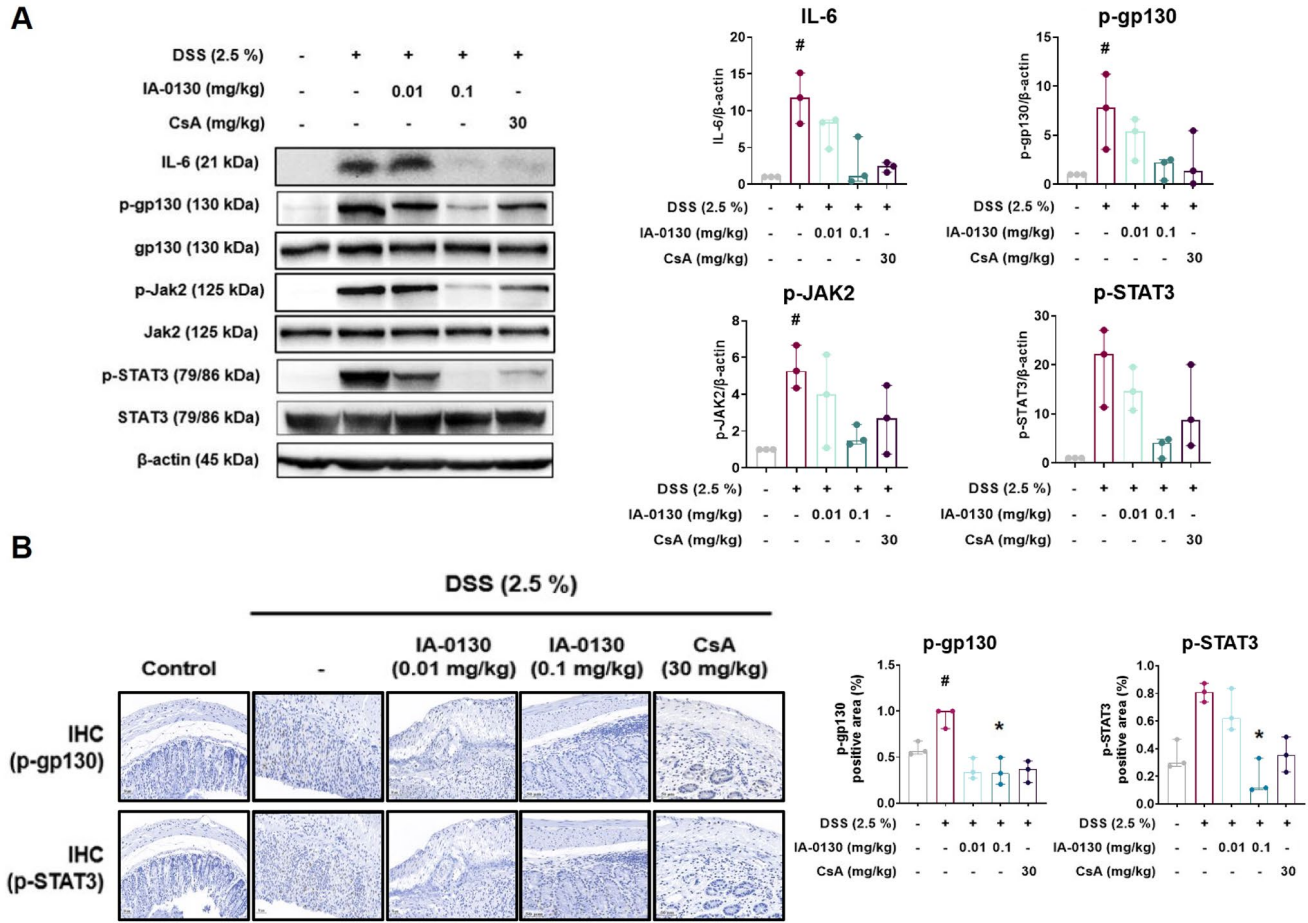
IA-0130 inhibited hyperactivation of IL-6 signaling in mouse colon tissues. IL-6-induced signal pathway in the colon tissues of mice, n = 3/group (A). IHC analysis of the expression of p-gp130 and p-STAT3 in the colon tissues of mice, n = 3/group (scale bar = 200 µm) (B). The data of Western blotting and IHC analysis were analyzed using Kruskal–Wallis test followed by the Dunn’s multiple comparisons test and the data are presented as the median with interquartile range. # *p* < 0.05 vs. control group, * *p* < 0.05, ** *p* < 0.01, *** *p* < 0.005 vs. DSS group. IHC, immunohistochemistry; p-, phophorylated-

We hypothesized that the inhibitory effect of IA-0130 on DSS-induced colitis symptoms and abnormal expression of inflammatory cytokines and TJ proteins may occur as a result of IA-0130 regulating the excessive activation of the IL-6 signaling pathway. Therefore, the regulatory effect of IA-0130 on the IL-6 signaling pathway in DSS-induced colitis conditions was evaluated. The results of Western blotting showed that IL-6 expression (H_3_ = 11.62, N_1-5_ = 3, p = 0.09, Kruskal–Wallis test) and phosphorylation of gp130 (H_3_ = 8.293, N_1-5_ = 3, p = 0.22, Kruskal–Wallis test), JAK2 (H_3_ = 8.192, N_1-5_ = 3, p = 0.40, Kruskal–Wallis test), and STAT3 (H_3_ = 10.37, N_1-5_ = 3, p = 0.09, Kruskal–Wallis test) were inhibited by 0.1 mg/kg IA-0130 treatment (Fig. 4A). Additionally, IHC analysis was performed to investigate whether IA-0130 suppresses the expression of p-STAT3 and p-gp130 in inflamed mouse colonic tissues (Fig. 4B). Thus, treatment with 0.1 mg/kg IA-0130 significantly down-regulated the expression of p-gp130 (H_3_ = 10.83, N_1-5_ = 3, p = 0.04, Kruskal–Wallis test) and p-STAT3 (H_3_ = 11.27, N_1-5_ = 3, p = 0.02, Kruskal–Wallis test). Taken together, these results indicated that IA-0130 attenuated DSS-induced mouse colitis by regulating the IL-6 signaling pathway in inflamed colonic tissues.

## Discussion

This study aimed to investigate whether IA-0130 suppresses colitis development. Oral administration of IA-0130 ameliorated colitis symptoms and down-regulated the pro-inflammatory cytokines expression in colon tissues. Additionally, IA-0130 regulated the mouse intestinal barrier function and gp130 signaling pathways in colonic tissues.

Current treatments for IBD include surgery, biologics, and fasting. Corticosteroids and immunosuppressant are also used to suppress IBD symptoms [29]. However, the use of antibody-based drugs may induce the development of drug resistance due to immunogenicity, and treatment with steroids and immunosuppressant can lead to serious side effects, such as infection [30, 31]. Therefore, studies to develop drugs with similar efficacy, safety, and ease of use as existing treatments are being conducted. IA-0130 is a small-molecule compound that can be administered orally and has shown anti-inflammatory effects similar to CsA even at relatively low concentrations. We used CsA as a control drug to evaluate the effects of IA-0130 on DSS-induced mouse colitis. CsA is one of immunosuppressive drug that can effectively suppress the progression of various immune diseases and is used to treat patients with IBD who are at risk of side effects due to long-term steroid administration [32]. However, since CsA is used as an immunomodulator, its use is limited due to side effects such as tremors, paresthesia, malaise, headache, abnormal liver function tests, gingival hyperplasia, and hirsutism [33]. Therefore, various small molecule compounds are actively being developed to compensate for the shortcomings of conventional IBD therapies [34].

To assess the anti-inflammatory effects of IA-0130, we examined its inhibitory effects on the progression of DSS-induced mouse colitis. In a previous study, a SERM was shown to ameliorate DSS-induced colitis via the promotion of Mrc-1 positive anti-inflammatory macrophage phenotype and regulation of pro-inflammatory cytokine and chemokine expression [35]. However, the efficacy of oxindoles for colitis treatment is unknown. Therefore, we investigated whether the 3-(1,3-diarylallylidene)oxindoles derivative IA-0130 exhibited anti-inflammatory effects on DSS-induced mouse colitis. Interestingly, treatment with 0.1 mg/kg IA-0130 significantly suppressed colitis symptoms such as weight loss and colon length shortening and restored mucosal destruction in mice with DSS-induced colitis (Fig. 1). This is the first study to demonstrate the suppressive effect of the oxindole derivative IA-0130 on the development of DSS-induced acute mouse colitis.

Various factors affect IBD pathogenesis, and inflammatory cytokines play an important role in the acute phase inflammation of colitis [14]. Additionally, pro-inflammatory cytokines impair intestinal barrier function, inducing apoptosis and reducing the proliferation of intestinal epithelial cells [15]. Therefore, cytokine regulation is important in the treatment of colitis. To verify our hypothesis that IA-0130 suppresses the excessive expression of pro-inflammatory cytokines, we analyzed the mRNA and protein expression of pro-inflammatory cytokines, TNF-α, IL-1β, IL-6, and IL-17A, which are important in colitis development and maintenance. We observed that IA-0130 significantly suppressed the overexpression of pro-inflammatory cytokines in inflamed mouse colonic tissues (Fig. 2). This may be because IA-0130 regulated the abnormal activation of immune cells such as T cells and macrophages in mouse colitis. To further verify this hypothesis, studies on the mechanism and effects of IA-0130 on immune cell population analysis will be needed.

Disruption of the intestinal barrier function is one of the causes of IBD, and various cytokines induce the activation of intestinal epithelial cells and affect barrier permeability. In a previous study, IL-6 was shown to increase the expression of the transcription factor CDX2 and claudin-2 via the gp130/mitogen-activated protein kinase kinase/phosphoinositide 3-kinase signaling pathway and upregulate permeability in Caco-2 intestinal epithelial cell monolayers [36]. Additionally, claudin-2 protein expression is increased in the mucosa of patients with UC, and claudin-2 overexpression plays an important role in intestinal barrier disruption [37]. Therefore, regulation of the TJ protein expression is important for maintaining the intestinal epithelial barrier functions. Based on the correlation between the cytokines and intestinal epithelial barrier function, we investigated whether IA-0130 suppresses the loss of barrier function by inhibiting the direct binding of gp130. We found that IA-0130 inhibited the DSS-induced increase in intestinal barrier permeability and effectively regulated the expression of TJ proteins (Fig. 3). These results demonstrated that IA-0130 effectively protected intestinal epithelial barrier function.

An increasing number of studies show that the development of colitis induces IL-6 overexpression and excessive JAK and STAT phosphorylation in colonic tissues [38]. In addition, activation of STAT3 promotes inflammation by enhancing the activation and survival of immune cells [39]. However, mice with *Stat3* knockdown show increased susceptibility to DSS and decreased epithelial integrity compared to wild-type mice [40]. STAT3 plays an important role in maintaining intestinal homeostasis at a steady state, but when overactivated, it also promotes inflammation. Therefore, it is important to suppress the hyperactivation of the intestinal IL-6 signaling pathway caused by colitis and regulate it to steady-state levels. In our previous study, IA-0130 was found to directly target gp130 and inhibit the activation of downstream signaling pathways in an ovarian cancer cell line. Therefore, we investigated whether IA-0130 suppresses the DSS-induced hyperactivation of the IL-6 signaling pathway in mouse colonic tissues in this study. Western blotting and IHC results showed that administration with IA-0130 inhibited the excessive activation of the gp130 signaling pathway in mouse colon tissues (Fig. 4). In conclusion, our results showed evidence for the preventive effects of IA-0130 on colitis development via the regulation of IL-6 signaling pathways.

There are limitations on this study. To evaluate the mechanism of action of IA-0130, which prevents the development of colitis by inhibiting the interaction between gp130 and IL-6, a more direct comparison would be possible if a small molecule or antibody specifically targeting gp130 was used as a control drug. Additionally, if the mouse group administered only IA-0130 had been included in the experimental group, independent effects that could have occurred only by administering the IA-0130 drug could have been confirmed. Additionally, if the group of mice administered only IA-0130 had been included in the experimental group, it would have been possible to monitor the independent effects that could occur only with the administration of IA-0130 drug. In addition, in experiments using mouse intestinal tissue collected immediately after mouse euthanasia, statistical analysis of variables over time was not performed, so one-way ANOVA was performed instead of two-way ANOVA. Identifying changes in the expression of factors according to DSS and drug administration time may also be meaningful in evaluating the efficacy of IA-0130 in more detail.

IA-0130 effectively inhibited the pathogenesis of colitis by modulating pro-inflammatory cytokine expression, protecting the intestinal epithelial barrier function to maintain intestinal barrier permeability, and inhibiting the hyperactivation of the IL-6 signaling pathway in DSS-induced mouse colitis. Our results demonstrated the potential of IA-0130 as an effective drug for the treatment of IBD and other immune diseases. Further studies investigating the efficacy and mechanism of IA-0130 on various immune disease and cancer models are required.

### Supplementary Information

Below is the link to the electronic supplementary material.

Supplementary file1 (PDF 410 KB)

Supplementary file2 (PDF 441 KB)

Supplementary file3 (PDF 817 KB)

Supplementary file4 (DOCX 27 KB)

Supplementary file5 (DOCX 28 KB)

## Data Availability

The datasets generated during and/or analyzed during the current study are available from the corresponding author upon reasonable request.

## Abbreviations

CsA: Cyclosporin A
DSS: Dextran sulfate sodium
gp130: Glycoprotein 130
IHC: Immunohistochemistry
IL-6: Interleukin-6
SERM: Selective estrogen receptor modulator
TJ: Tight junction
