# Supplementary material for: A derivative of 3-(1,3-diarylallylidene)oxindoles inhibits dextran sulfate sodium-induced colitis in mice
Source: Pharmacol Rep. 2024 Jun 25;76(4):851–62. doi: 10.1007/s43440-024-00616-2 (PMC11294400; doi:10.1007/s43440-024-00616-2)

Fig. 1S Whole membranes of blots in Western blotting

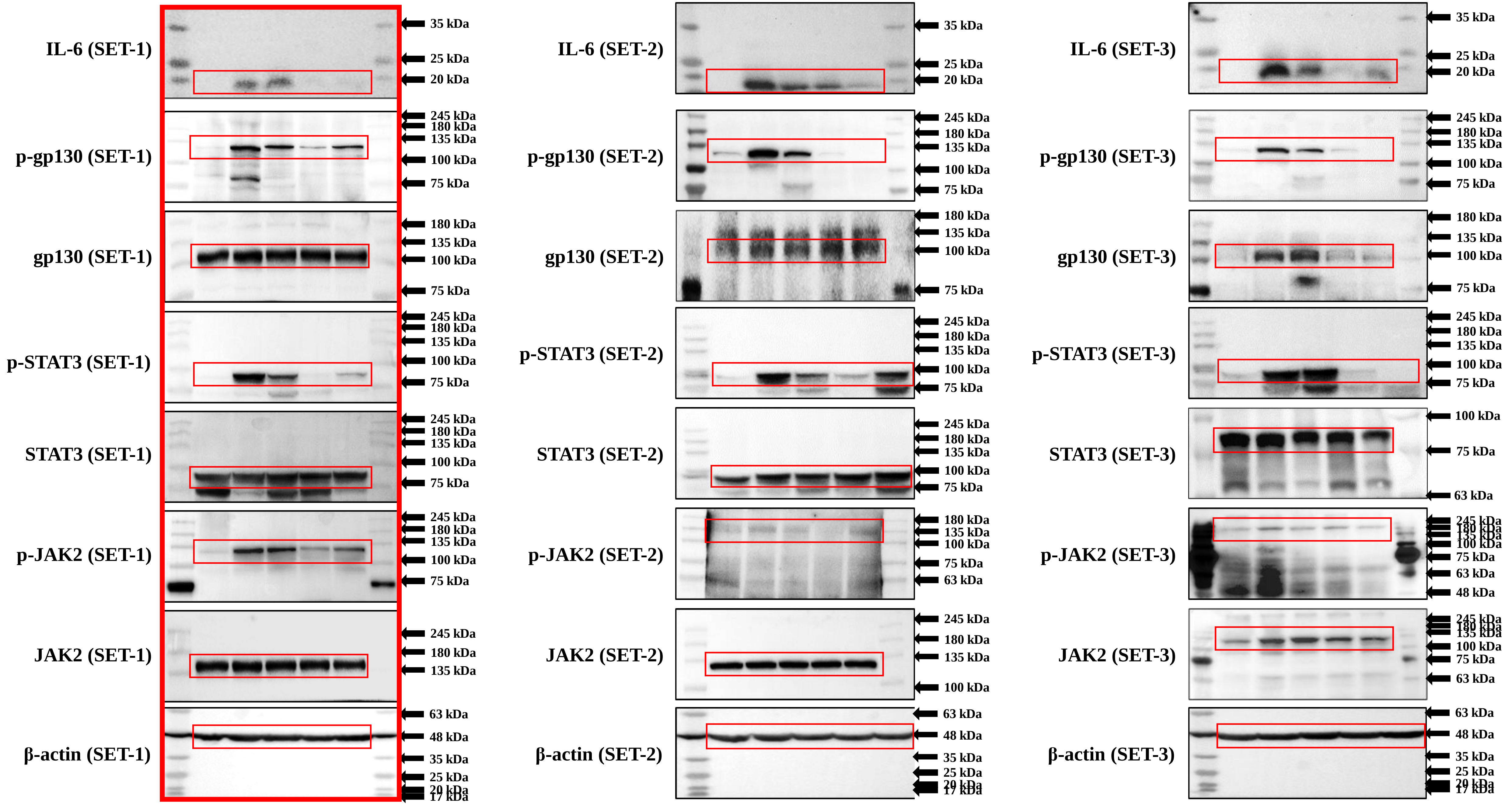

Fig. 1S Whole membranes of blots in Western blotting

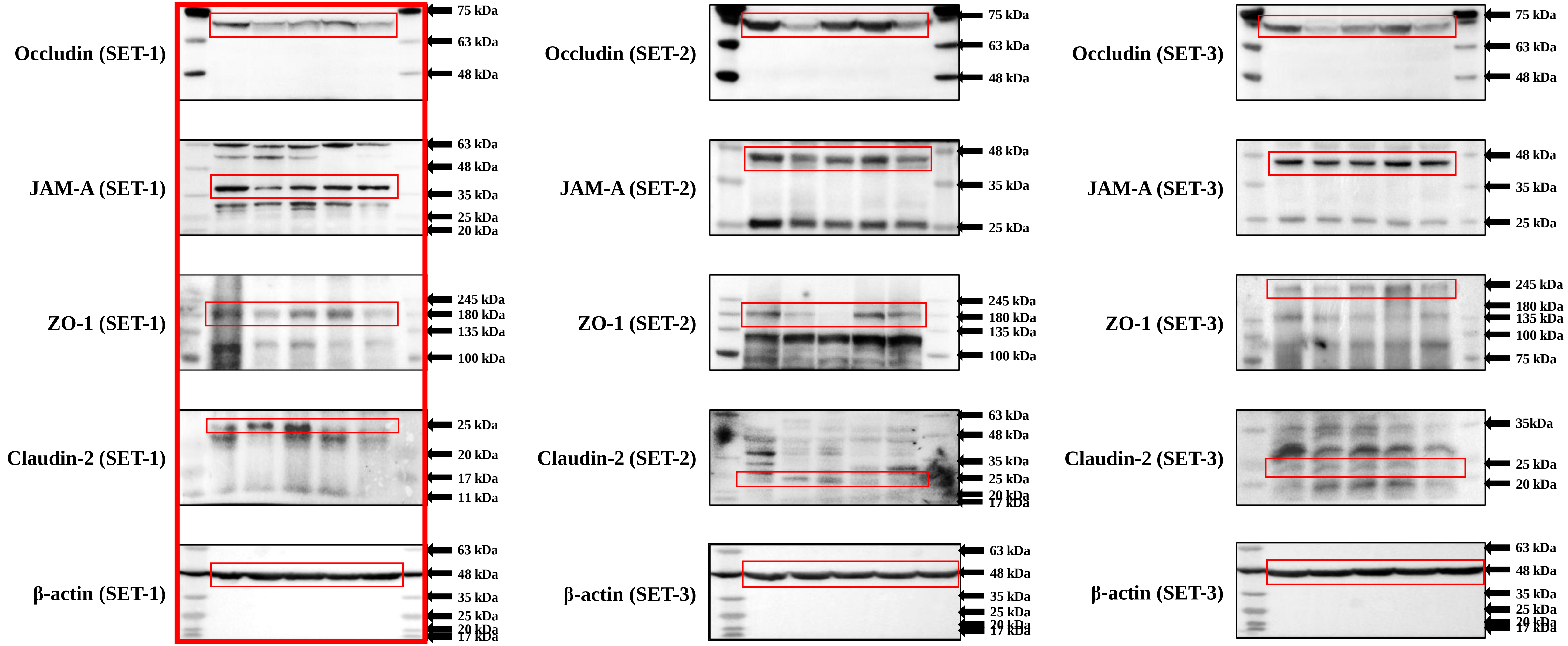

Supplement: Supplementary file 1 — Supplementary file1 (PDF 410 KB) [file 43440_2024_616_MOESM1_ESM.pdf]
