# Supplementary material for: A derivative of 3-(1,3-diarylallylidene)oxindoles inhibits dextran sulfate sodium-induced colitis in mice
Source: Pharmacol Rep. 2024 Jun 25;76(4):851–62. doi: 10.1007/s43440-024-00616-2 (PMC11294400; doi:10.1007/s43440-024-00616-2)
Supplement: Supplementary file 2 — Supplementary file2 (PDF 441 KB) [file 43440_2024_616_MOESM2_ESM.pdf]

Fig. 2S Representative images of hematoxylin and eosin (H&E) staining (Fig. 1E)

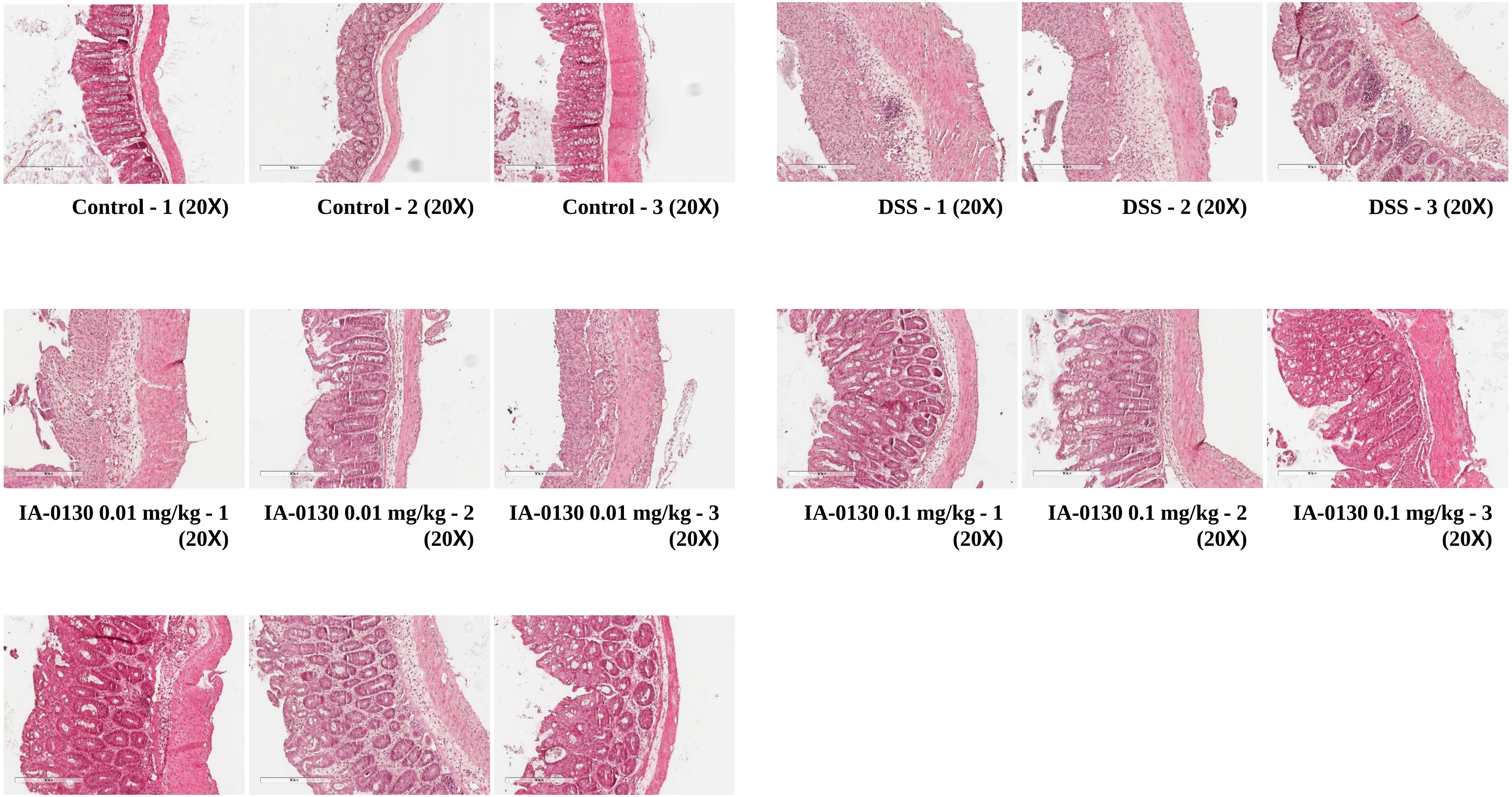

Control - 1 (20X) | Control - 2 (20X) | Control - 3 (20X) | DSS - 1 (20X) | DSS - 2 (20X) | DSS - 3 (20X)

IA-0130 0.01 mg/kg - 1 (20X) | IA-0130 0.01 mg/kg - 2 (20X) | IA-0130 0.01 mg/kg - 3 (20X) | IA-0130 0.1 mg/kg - 1 (20X) | IA-0130 0.1 mg/kg - 2 (20X) | IA-0130 0.1 mg/kg - 3 (20X)

CsA 30 mg/kg - 1 (20X) | CsA 30 mg/kg - 2 (20X) | CsA 30 mg/kg - 3 (20X)
