# Supplementary material for: A derivative of 3-(1,3-diarylallylidene)oxindoles inhibits dextran sulfate sodium-induced colitis in mice
Source: Pharmacol Rep. 2024 Jun 25;76(4):851–62. doi: 10.1007/s43440-024-00616-2 (PMC11294400; doi:10.1007/s43440-024-00616-2)
Supplement: Supplementary file 3 — Supplementary file3 (PDF 817 KB) [file 43440_2024_616_MOESM3_ESM.pdf]

Fig. 3S-(1) Representative images of immunohistochemistry (IHC) staining, p-gp130 (Fig. 4B)

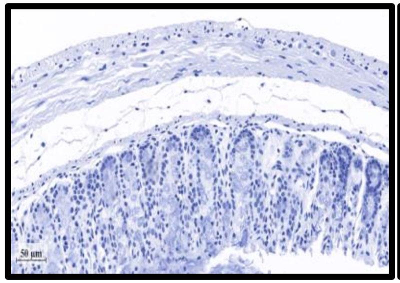

Control - 1

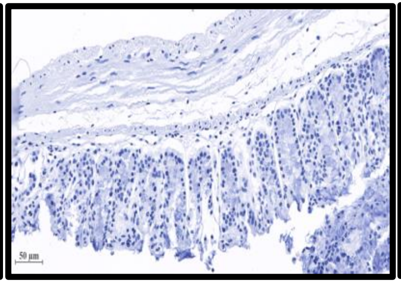

Control - 2

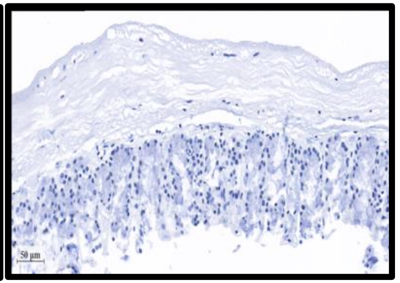

Control - 3

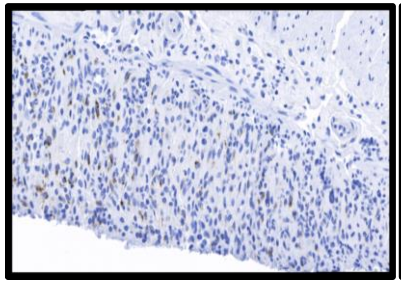

DSS - 1

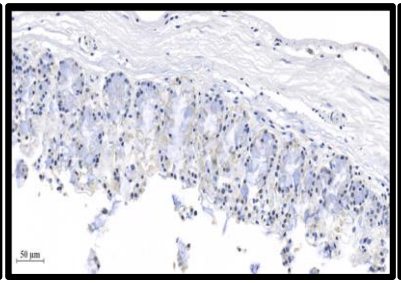

DSS - 2

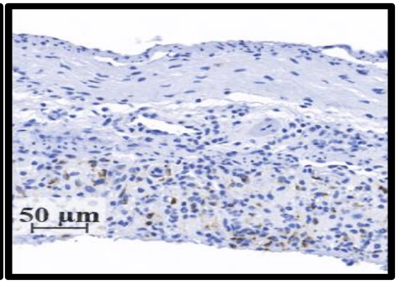

DSS - 3

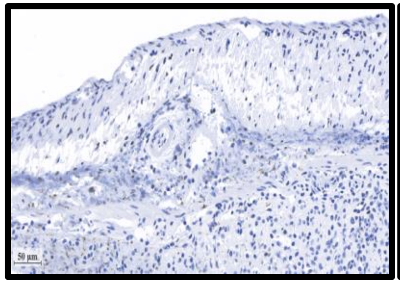

IA-0130 0.01 mg/kg - 1

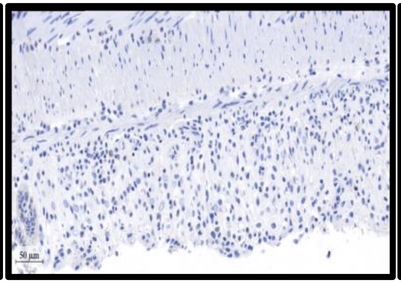

IA-0130 0.01 mg/kg - 2

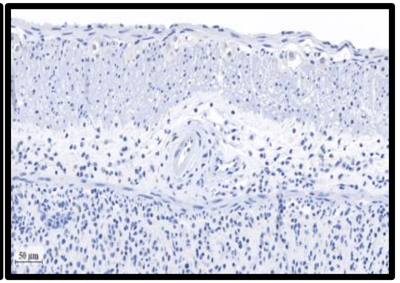

IA-0130 0.01 mg/kg - 3

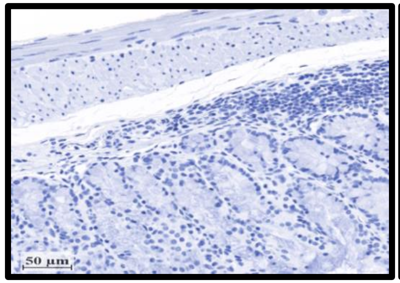

IA-0130 0.1 mg/kg - 1

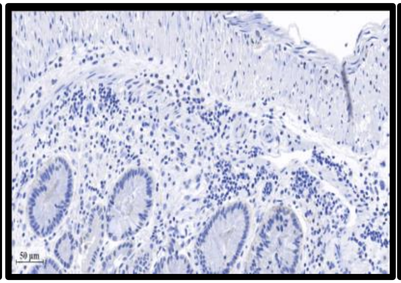

IA-0130 0.1 mg/kg - 2

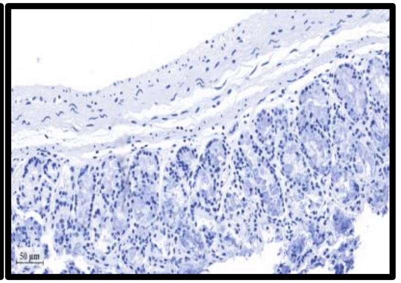

IA-0130 0.1 mg/kg - 3

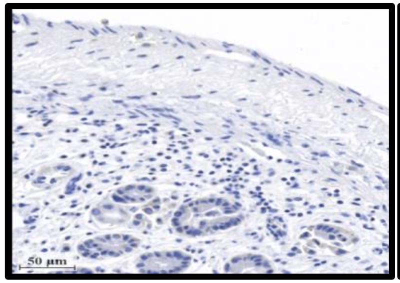

CsA 30 mg/kg - 1

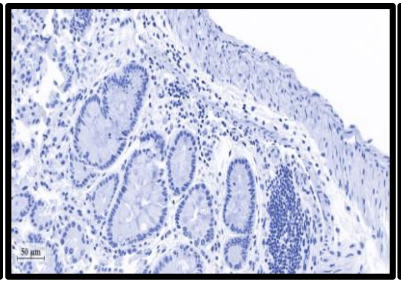

CsA 30 mg/kg - 2

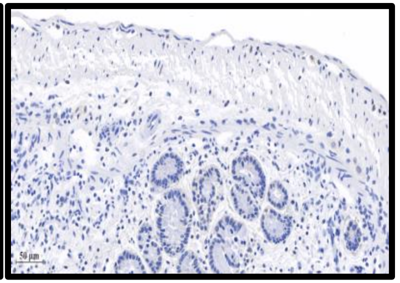

CsA 30 mg/kg - 3

Fig. 3S-(2) Representative images of immunohistochemistry (IHC) staining, p-STAT3 (Fig. 4B)

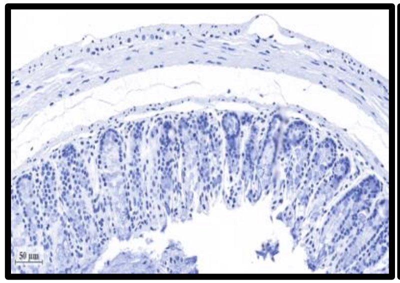

Control - 1

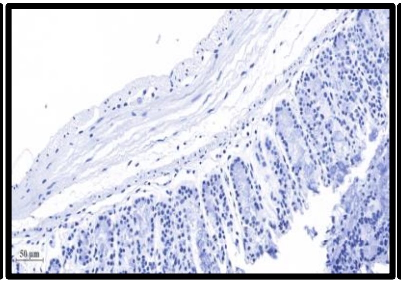

Control - 2

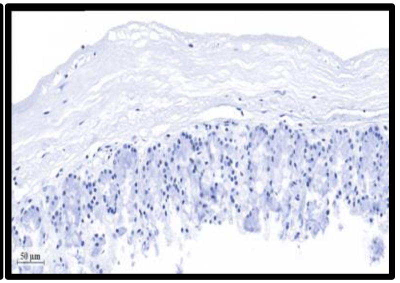

Control - 3

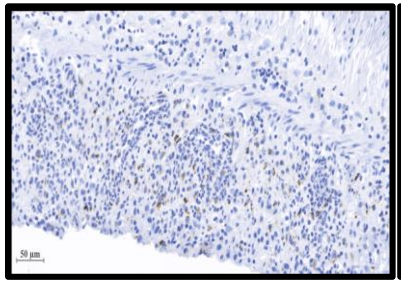

DSS - 1

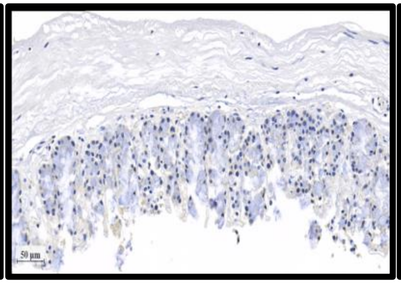

DSS - 2

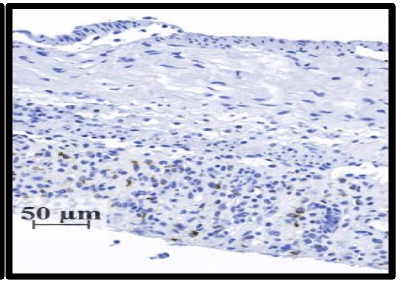

DSS - 3

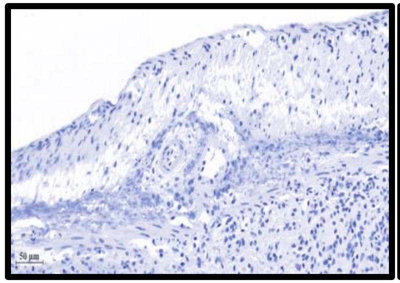

IA-0130 0.01 mg/kg - 1

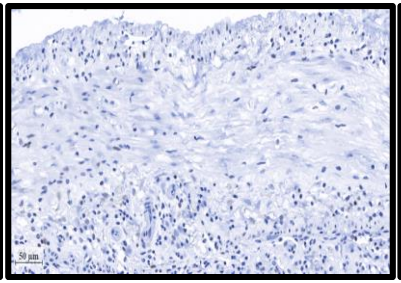

IA-0130 0.01 mg/kg - 2

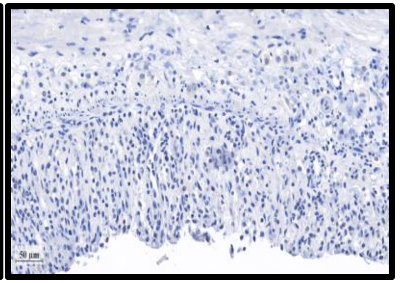

IA-0130 0.01 mg/kg - 3

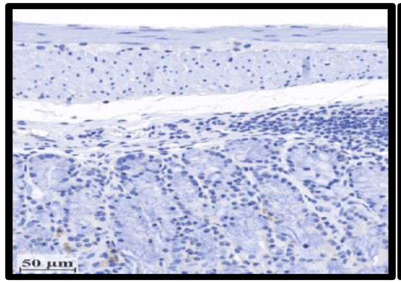

IA-0130 0.1 mg/kg - 1

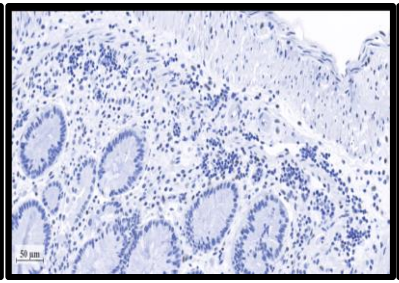

IA-0130 0.1 mg/kg - 2

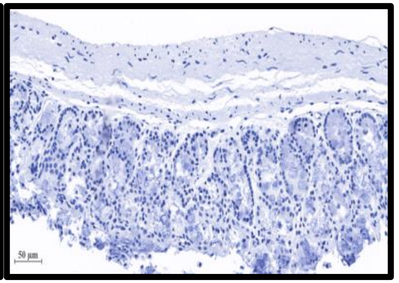

IA-0130 0.1 mg/kg - 3

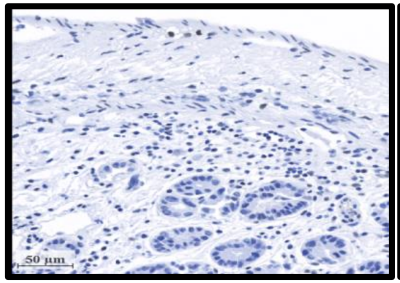

CsA 30 mg/kg - 1

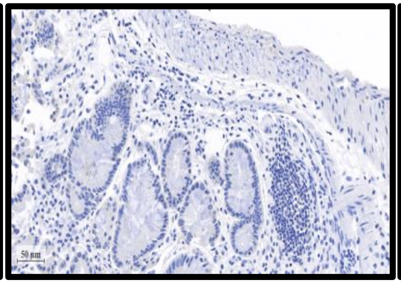

CsA 30 mg/kg - 2

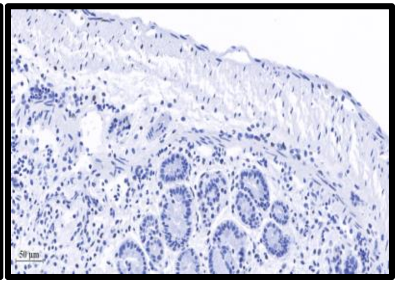

CsA 30 mg/kg - 3
