# Supplementary material for: A derivative of 3-(1,3-diarylallylidene)oxindoles inhibits dextran sulfate sodium-induced colitis in mice
Source: Pharmacol Rep. 2024 Jun 25;76(4):851–62. doi: 10.1007/s43440-024-00616-2 (PMC11294400; doi:10.1007/s43440-024-00616-2)
Supplement: Supplementary file 4 — Supplementary file4 (DOCX 27 KB) [file 43440_2024_616_MOESM4_ESM.docx]

**Table S1. Disease activity index (DAI) scoring system.**

| **Score** | **Weight loss** | **Stool consistency** | **Bleeding** |
| --- | --- | --- | --- |
| 0 | None | None | None |
| 1 | 1 – 5 % | Loose stools | None |
| 2 | 5 – 10 % | Loose stools | Slightly bleeding |
| 3 | 10 – 15 % | Loose stools | Gross bleeding |
| 4 | >15 % | Diarrhea | Massive bleeding |
