# Supplementary material for: A derivative of 3-(1,3-diarylallylidene)oxindoles inhibits dextran sulfate sodium-induced colitis in mice
Source: Pharmacol Rep. 2024 Jun 25;76(4):851–62. doi: 10.1007/s43440-024-00616-2 (PMC11294400; doi:10.1007/s43440-024-00616-2)
Supplement: Supplementary file 5 — Supplementary file5 (DOCX 28 KB) [file 43440_2024_616_MOESM5_ESM.docx]

**Table S2. Histological scoring system of dextran sulfate sodium-induced colitis in mouse**

| **Score** | **Histological feature** | **Description** |
| --- | --- | --- |
| 0 | Loss of epithelium | None |
| 1 |  | 0-5 % loss of epithelium |
| 2 |  | 5-10 % loss of epithelium |
| 3 |  | Over 10 % loss of epithelium |
| 0 | Crypt damage | None |
| 1 |  | 0-10 % loss of crypt |
| 2 |  | 10-20 % loss of crypt |
| 3 |  | Over 20 % loss of crypt |
| 0 | Depletion of goblet cells | None |
| 1 |  | Mild depletion |
| 2 |  | Moderate depletion |
| 3 |  | Severe depletion |
| 0 | Infiltration of inflammatory cells | None |
| 1 |  | Mild infiltration |
| 2 |  | Moderate infiltration |
| 3 |  | Severe infiltration |
